# Employee creativity and innovation in higher education institutions: applying the dynamic componential model of creativity and innovation

**DOI:** 10.3389/fpsyg.2025.1614751

**Published:** 2025-10-13

**Authors:** Azadeh Amoozegar, Oghenekevwe Erasmos Esohwode, Wang Yujiao, Wen Hui Pee, Ayman Ismaeil, Mohit Yadav, Mohd Taib Harun

**Affiliations:** ^1^Faculty of Education and Liberal Arts, INTI International University, Nilai, Malaysia; ^2^Limkokwing Graduate School, Limkokwing University of Creative Technology, Cyberjaya, Malaysia; ^3^School of Management Engineering, Zhejiang Guangsha Vocational and Technical University of Construction, Dongyang City, Zhejiang Province, China; ^4^Records and Archives Management Department, College of Business Administration, A'Sharqiyah University, Ibra, Oman; ^5^Jindal Global Business School, O.P. Jindal Global University, Sonipat, Haryana, India

**Keywords:** inclusive education system, educational policies, developing countries, R & D investment, process innovation

## Abstract

**Introduction:**

In today's highly competitive landscape, higher education institutions must adopt innovative practices to thrive and maintain academic excellence. The engagement of academic staff is essential for driving such innovation. While previous studies have examined the influence of personal and workplace factors separately, this study investigates the roles of employee resilience, emotional intelligence, and job autonomy—alongside the mediating effect of employee creativity—on employee innovation in higher education, drawing on the Dynamic Componential Model of Creativity and Innovation.

**Methods:**

A quantitative research design was used, surveying 307 academic staff members from federal and state universities in South-Western Nigeria. The study employed a structural equation modeling approach via SmartPLS4 to test the hypothesized relationships among employee resilience, emotional intelligence, job autonomy, creativity, and innovation.

**Results:**

The findings confirm that employee resilience, emotional intelligence, and job autonomy significantly contribute to employee innovation. Additionally, mediation analysis reveals that both resilience and job autonomy foster innovation indirectly through employee creativity, whereas emotional intelligence does not exert a significant indirect effect.

**Discussion:**

These results underscore the importance of cultivating resilience, autonomy, emotional intelligence, and creativity among academic staff as a strategic means to promote innovation within universities. They demonstrate that interventions emphasizing both personal growth and supportive work environments can meaningfully advance innovative practices and enhance academic excellence.

## 1 Introduction

In higher education, globalization has been a game changer since the twentieth century (Black et al., 2019). Higher education as the great society equalizer has evolved into a contentious arena where diverse interests now compete for influence (Asmamaw and Semela, 2023). In response to fierce competition and the pressures of the global market, institutions are compelled to seek out innovative approaches to reinvent themselves and sustain their competitive position (AlEssa and Durugbo, 2021). In today's global academic landscape, universities face intense competition in rankings, thus, it is crucial for these institutions to leverage their staff's innovative capabilities as a strategic tool to accomplish their objectives (Jibola, 2020). Nöhammer and Stichlberger (2019) assert that to succeed in today's dynamic business environment, institutions must actively pursue innovation to maintain a sustainable competitive advantage. As noted by Dixit and Upadhyay (2021), fostering innovation is essential for establishing and preserving a durable competitive advantage. The literature in this field has shown that workplace innovation significantly contributes to an organization's performance and efficiency (Corzo Morales and Contreras Pacheco, 2024).

To succeed in the constantly evolving landscape of education, universities require their employees to demonstrate innovative work behavior as an essential component (Namono et al., 2024). Innovative work behavior is a crucial professional requirement for education institutions to survive in the challenging dynamic environment and keep up to date with the rapidly changing societal demands (Namono et al., 2022). This dynamism requires organizations to recruit employees who can resiliently adjust their work roles to align with evolving environmental conditions (Bhamra, 2015). Innovative individuals possess the resilience to embrace risks, enabling them to devise and implement novel occupational roles that are pertinent to society (Mafabi et al., 2015). Innovative work behavior encompasses a multifaceted set of actions aimed at discovering, creating, advocating for, and executing novel concepts within the workplace environment (Luthans and Youssef-Morgan, 2017; Wang et al., 2022). Within academic environments, innovation can be understood as the capacity of staff members to engage in creativity activities. This involves embracing, developing, and implementing novel approaches to fulfill their professional responsibilities. Such innovative practices may encompass areas like research methodologies, instructional techniques, and the invention and application of cutting-edge technologies (Namono et al., 2022).

The educational development in Africa, particularly in Nigeria, exhibits a comparable trajectory to that of more developed nations, albeit progressing at a considerably slower rate (Ugwu, 2024). Nigeria's goal of becoming one of the top 20 economies in the world might not be realized unless the nation leverages its vast resources through education (Nwanshak et al., 2021). In Nigeria, the government has recognized higher education, particularly at the tertiary level, as a crucial component in its efforts to achieve the objectives outlined in the National Economic Empowerment and Development Strategies (NEEDS). This emphasis on education is seen as fundamental to the country's developmental aspirations (Olutola and Olatoye, 2015). According to the National Policy on Education in Nigeria [National Education Research and Development Council (NERDC), 2013], tertiary education encompasses universities, polytechnics, and colleges of education, as well as affiliated institutions (Adefulu et al., 2020). Higher education institutions in Nigeria are dedicated to the core functions of instruction, scientific inquiry, and public outreach. These activities aim to cultivate human capital and disseminate essential knowledge required by various sectors, including industry (Adefulu et al., 2020).

The landscape of higher education is multifaceted, demanding the most innovative minds to ensure its effective operation (Castells, 1994). As noted by Bawuro et al. (2019), higher education plays a crucial role in fostering economic growth through innovative and creative ideas that benefit both businesses and governments. Higher Education is a widely sought-after resource that significantly contributes to the development of global leaders, industry executives, educators, medical professionals, engineers, and experts across nearly every sector of society (Bawuro et al., 2019). The advancement of a nation is intrinsically linked to university education, which serves as a cornerstone for producing the high-level workforce essential for economic progress and effective governance. By cultivating skilled professionals, tertiary education equips countries with the human capital necessary to drive economic growth and manage societal affairs competently (Jibola, 2020). This perspective on education holds true in Nigeria; however, the actual experience falls short of expectations (Ugwu, 2024) and over 60% of university graduates in Nigeria are missing the fundamental skills that employers desire (Ojayeola et al., 2018). This shortfall can be attributed to the inability of academic staff to demonstrate innovative performance, resulting in universities failing to produce creative graduates for the job market (Namono et al., 2022).

Contrary to the typical role of universities as centers for research and innovation (Namono et al., 2024), Nigerian universities have fallen short in this regard. The Nigerian higher education system seems to be unable to foster innovative and creative thinking among its academic personnel (Agboola and Ukoette, 2019). Educators there report a lack of supportive system necessary to foster innovation within the academic sphere (Bawuro et al., 2019). Nigeria's university system has encountered various obstacles related to its administration and management (Aluede et al., 2012). These challenges include insufficient staff benefits, limited opportunities for professional growth (Adeyemo Adeyinka et al., 2013; Akinduyo, 2014; Isa and Yusoff, 2015), inadequate infrastructure, overpopulated classrooms, weak research and innovation capabilities, outdated course content, and the exodus of skilled academics (Ayo-Odifiri, 2023; Ezepue and Ochinanwata, 2017; Umoru, 2020). Continuous strikes, salary shortfalls, and promotion delays have widened the gap between education and instruction (Bawuro et al., 2019). Feeling undervalued, academic staffs often convey their dissatisfaction to students through apathetic teaching, inadequate support for academic struggles, and insufficient mentoring. When university management and government officials support faculty endeavors and demonstrate appreciation, lecturers are more inclined to impart innovative skills to students through innovative teaching. Subsequently, students apply this creative knowledge in their professional environments by executing tasks in an innovative and creative manner (Thurlings et al., 2015). Hence, as centers of innovation, universities require their staff to be innovative to stay current with advancements in education (Ahmad, 2020).

As noted, higher education is considered crucial for a nation's socioeconomic progress by developing its human resources into productive members of society (Jibola, 2020). The innovative behavior of university lecturers enable the higher education sector to adapt to societal changes and boost their self-confidence, information exchange, creative thinking, and human potential in academic instruction, scientific inquiry, and public service initiatives (Uzochukwu et al., 2016). Ezeh et al. (2020) define innovative behavior as a deliberate action by an individual to introduce and implement novel ideas within an organization to enhance its overall performance. Bawuro et al. (2019) argue that such behaviors form a crucial foundation for addressing and managing emerging challenges to gain a competitive edge. Research has shown that organizations can better navigate the challenges of a dynamic and competitive business environment when their employees are capable of engaging in innovative work practices, which include generating, adopting, and implementing new ideas for products and work processes (Muchiri et al., 2020). Consequently, it is imperative for educational systems to cultivate creativity and innovation among employees, enabling them to develop effective solutions to address various challenges.

In our current globalized society, which is reshaping our lifestyle and business practices, innovation plays a crucial role (Schaap, 2017). Higher education institutions often struggle to keep pace with environmental changes, despite the necessity to address these new circumstances (Morgan, 2020). Educational organizations that fail to adapt and generate innovative solutions risk facing severe consequences (Crow and Dabars, 2015). To thrive in rapidly evolving and dynamic environments, organizations frequently depend on their employees' creativity and their capacity to transform creative ideas into innovative actions (Anderson et al., 2014; Nisula, 2013). Although creativity and innovation are often mentioned together (Kimwolo and Cheruiyot, 2019), they have distinct characteristics. Creativity is associated with generating novel and valuable concepts, whereas innovation pertains to the practical application of these ideas (Alt et al., 2023; Laud et al., 2023). Prior scholars argue that innovative work behavior is a sequential construct, with creativity serving as the initial phase in the innovation process. This process encompasses three stages: idea exploration, idea generation, and idea championing. Idea exploration refers to an individual's capacity to examine a problem and conceive novel approaches to resolve it. Idea generation involves an individual's ability to produce alternative solutions for addressing work-related issues. Lastly, idea championing pertains to an individual's skill in persuading and convincing colleagues to support the newly developed ideas (Yuan and Woodman, 2010). Therefore, creativity serves as the initial phase in the innovation process, encompassing the exploration, generation and promotion of new ideas. In contrast, innovation extends beyond idea generation to include the implementation of these concepts to create value (Namono et al., 2022). Given that creativity and innovation involve distinct activities, it is essential to examine more closely how creativity can function as a mediating factor in this study, bridging the gap between elements that influence innovative work behavior.

Amidst the global competition in university rankings, it is crucial for institutions to capitalize on the innovative tendencies of their staff to fulfill their strategic goals (Jibola, 2020). In higher education, the drive for innovation has become essential as institutions strive to respond to swiftly changing challenges and take advantage of new opportunities. Namono et al. (2022) mentions the need for university employees to be innovative “to keep up with educational innovation.” This highlights the unique demands of the higher education sector. Given the distinctive nature of higher education, which focuses on instruction, research, and societal involvement, more research is needed to understand how innovative work behaviors manifests itself specifically within academic settings and what unique factors influence it. Much of the existing research on innovative behavior draws from studies in business and industry settings (Ezeh et al., 2020; Ibeku and Nwagwu, 2024; Olayemi et al., 2020). There is lack of research about staff innovative behavior in Nigeria University (Bawuro et al., 2019; Dahiru and Opeyemi, 2020; Jibola, 2020). Consequently, this research aims to address the shortcomings left by earlier researchers.

This study makes three significant contributions to existing literature. First, it adds to the body of knowledge on innovation within educational contexts. Research on innovation is crucial for universities, as they educate students who will apply their skills across various career fields (Dehning et al., 2020; Mukyala and Namono, 2024). Second, this research makes a theoretical contribution to the dynamic componential model of creativity and innovation (Amabile and Pratt, 2016) by examining the degree to which individual factors influence creativity and innovative work behavior. While most research on creativity and innovative work behavior has employed social exchange theory (Bawuro et al., 2019; Uppathampracha and Liu, 2022; Volery and Tarabashkina, 2021) and social cognitive theory (Karaboga et al., 2022; Namono et al., 2024, 2022), few studies have utilized the dynamic componential model of creativity and innovation. Thirdly, the research demonstrates how employee creativity serves as a mediator between individual factors such as resilience and innovative work behavior, utilizing empirical data from educational environments. Namono et al. (2024), point out that research on innovation in the service sector is limited, and previous studies examining employees' innovative work behavior in the education field have rarely investigated the simultaneous relationships among job autonomy, emotional intelligence, employee resilience, and innovative work behavior. The paper is structured into four main parts following this introduction. A review of relevant literature is presented in Section 2. Section 3 outlines the research methodology, including data gathering techniques, procedural steps, sampling methods, and the validity and reliability of the research instruments. The findings from the analysis are detailed in Section 4. The final section, Section 5, offers a discussion of the results, concluding remarks, highlights the study's contributions, and proposes recommendations for future research.

## 2 Theoretical framework and hypothesis development

The Dynamic Componential Model of Creativity and Innovation, developed by Amabile and Pratt (2016) provides a comprehensive framework that elucidates how workplace environment can nurture creative thinking and innovative practices among employees. The dynamic componential model keeps the original model's componential structure while adding a variety of additional dynamic aspects, such as feedback loops. These loops provide not just ways to launch future iterations through the creative process, but also mechanisms by which those future iterations could be different from prior ones. The major changes that Amabile and Pratt (2016) made fall into two high-level categories: new linkages between innovation and creativity, and new critical psychological factors. The model combines the organizational innovation process with the individual creative process, establishing several essential links between them. The results of creativity from individuals or small groups are integrated into the innovation process. At this stage, the association indicates that creativity is not actually the “fuzzy front end” of innovation; rather, it aligns more closely with the “fuzzy middle segment” (Amabile and Pratt, 2016).

The psychological and social elements that inspire employees to be creative are the main emphasis of the componential theory (Shafait and Huang, 2023). In higher education institutions, when both employees and organizations cultivate a mutual understanding of psychological and social wellbeing, employees are more inclined to engage in innovative activities. According to the Dynamic Componential Model of Creativity and Innovation (Amabile and Pratt, 2016), individual characteristics are essential in promoting creativity and innovative behavior among employees. While the significance of both organizational and personal factors was highlighted over 20 years ago (Amabile et al., 1996), the majority of research has concentrated on organizational precursors. There is a common belief that fostering employee innovation involves implementing various organizational elements that intentionally encourage staff to generate new ideas, address challenges, and seize opportunities. This perspective, however, overlooks the impact of individual factors that can also drive innovation, resulting in an incomplete understanding of the elements organizations should utilize (Volery and Tarabashkina, 2021). Although research on the factors leading to employee innovation is expanding, investigations into individual-level determinants remain limited. This research explores the influence of personal factors to address these limitations and enhance our comprehension of innovative work behavior. The dynamic componential model is expected to inspire valuable new empirical studies, which will enhance our comprehension of creativity and innovation. Additionally, it will offer a novel set of guidelines for organizational leaders aiming to foster innovation within their sectors.

### 2.1 Innovation in higher education

The critical role of innovation in an organization's success and longevity is well-recognized (Uppathampracha and Liu, 2022). Over the past twenty years, researchers and business experts have shown increasing attention to the idea of employee innovative work behavior (IWB) (Volery and Tarabashkina, 2021). This heightened focus stems from the constant pressure organizations face to innovate their products and services in order to stay competitive (Morgan, 2020; Zacher and Rosing, 2015). The driving force behind this ongoing innovation is ultimately the individuals within these organizations (De Jong and Den Hartog, 2007). Consequently, it is not unexpected that scholars are increasingly focusing on innovative behaviors at the individual level (Martín et al., 2018; Rigtering et al., 2019). Therefore, as employees are the cornerstone of an organization's innovative capabilities it is critical to understand the factors that can promote employees' innovative work behavior (Phairat and Potipiroon, 2022).

In today's dynamic educational landscape, universities, as hubs of knowledge, need to cultivate innovative work behavior among their employees to remain competitive (Namono et al., 2021). Innovation in higher education is characterized by the institution's ability to create and implement a new or significantly enhanced process, product, or organizational method that has a profound impact on the institution's operations and its stakeholders, such as students, communities, and businesses (Elrehail et al., 2018). An organization's ability to innovate is reflected in its employees, who are at the forefront in developing and integrating innovative ideas into their job functions or processes (Amabile and Pratt, 2016). However, the university workforce in Nigeria is perceived to have insufficient innovative capabilities, as evidenced by their struggles to embrace new work practices, conduct research, create new knowledge, and adapt to evolving methodologies. This deficiency hinders the transmission of innovative concepts to students. Therefore, this research aims to address this gap by identifying factors that can enhance the creativity and innovative behavior of academic personnel.

The significance of innovation for a nation's prosperity and the longevity of higher education institutions has led researchers to identify numerous personal and organizational factors that influence innovations in higher education institutions (Elrehail et al., 2018; Shafique et al., 2020), including organizational support, employee creativity, and work centrality (Volery and Tarabashkina, 2021), ethical leadership (Jibola, 2020; Uppathampracha and Liu, 2022), leader influence (Maulding, 2023), self-efficacy (Namono et al., 2024, 2022), work engagement (Mbuni, 2021), work environment (Hai et al., 2024), hope (Namono et al., 2021), prosocial motivation (Bawuro et al., 2019), transformational and authentic leadership (Elrehail et al., 2018), and high performance work systems (Phairat and Potipiroon, 2022). However, the effects of certain factors are still not fully understood, and there are numerous gaps in the existing research.

### 2.2 Emotional intelligence

Ideas form the foundation of innovation, and it is the individuals within organizations who generate and refine these ideas. Consequently, understanding the factors that drive employees' innovative behavior becomes crucial. Emotion stands out as one of these key factors, playing a crucial role in enhancing managerial effectiveness, improving job performance, and elevating the quality of innovations (Dasgupta, 2023). The dynamic componential model has highlighted the importance of emotions in creativity (Amabile and Pratt, 2016). Similarly, Malik (2021) discovered that individuals with high emotional intelligence were more likely to generate and implement innovative ideas in the workplace. Goleman (1998) suggests that emotional intelligence accounts for approximately 67% of the skills necessary for high performance. Consequently, in a knowledge-intensive organization, emotional intelligence could potentially steer individuals toward innovative work practices, thereby enhancing overall performance.

Emotions serve a crucial function in human life. Experiencing positive emotions can enhance an individual's immediate cognitive and behavioral repertoire, contributing to the formation of durable personal capabilities. Conversely, negative emotional states tend to constrict one's attentional focus (Liu et al., 2023). Recent studies highlight the significance of emotional intelligence as a key predictor in crucial areas such as academic settings, job performance, negotiation, leadership, emotional labor, trust, work–family conflict, and stress. However, there is a scarcity of literature on emotional intelligence within higher education institutions (HEIs), while in modern psychology, HEIs need emotional intelligence (Shafait and Huang, 2023). As a result, it is essential for researchers in the educational field to thoroughly investigate emotional intelligence to persuade educators of its benefits in managing difficult situations (Shafait et al., 2021). Emotional intelligence enables educators to effectively assess situational requirements and apply emotional strategies appropriately (Yin et al., 2013), thereby enhancing the quality of education and elevating societal success standards through Emotional intelligence (Zhoc et al., 2018).

The area of Emotional Intelligence (EI) has been explored by various researchers (Asmamaw and Semela, 2023; Batey and Furnham, 2006; Carroll, 2017; Hatamleh, 2021; Huntley, 2024; Jones, 2007; Mashi, 2023; Parker, 2019; Sembiring et al., 2020). EI is an ability that enables individuals to be aware of their emotional power and to control it as a connecting strength (Shafait et al., 2021). Emotional intelligence (EI) refers to a person's ability to understand and manage their own and others' emotions effectively (Asmamaw and Semela, 2023). Zhu et al. (2022) described emotional intelligence (EI) as either a cognitive ability, a trait, or a collection of skills and attributes related to how individuals perceive and integrate emotions, comprehend their own and others‘ feelings, and manage emotions to support their thoughts and actions. According to Udod et al. (2020), employees with high emotional intelligence are better equipped to understand their environment, inspire others, and generate positive emotions in people, which fosters engagement and innovation in the workplace. Emotional challenges are prevalent for employees at every organizational level (Alam and Singh, 2021); consequently, the persistent monitoring of emotional wellbeing and the execution of regular emotion evaluations have emerged as critical areas of focus for companies and scholars (Liu et al., 2023).

The emotional states of employees play a crucial role in fostering creativity (Seo, 2021). Fredrickson (2001) suggests that positive emotions can widen the scope of attention and cognitive processes, thereby encouraging flexible and creative thought. Lin et al. (2014) also found that positive emotions can enhance creative performance by fostering cognitive adaptability. Chong et al. (2020) conducted research that revealed a notable correlation between emotional intelligence and work performance among academic staff employed in private tertiary education institutions. The research conducted by Shafait et al. (2021) demonstrated that emotional intelligence has a positive influence on both knowledge management processes and creative output among university faculty members. According to Shafait and Huang (2023), emotional intelligence encourages individuals to recognize emotions, thereby altering their cognitive processes and behaviors, which ultimately leads to enhanced creativity and innovative work behavior. Hence, professionals should understand and maintain a clear perspective on EI within their organization, utilizing these concepts to enhance the overall creativity of both individual members and the higher education institution as a whole (Shafait et al., 2021). These results highlight the significance of emotional intelligence in multiple facets of tertiary education, such as creativity, innovation, and job performance. Drawing on the given arguments, this study proposes a significant relationship between emotional intelligence, employee creativity, and employee innovation.

*H1: Emotional Intelligence is significantly related to employee innovation*.
*H2: Emotional Intelligence is significantly related to employee creativity*.

### 2.3 Employee resilience

A crucial component of this research involves exploring resilience. The concept of employee resilience encompasses an individual's capacity to approach difficulties optimistically and enhance the understanding of a motivational mechanism (Anser et al., 2022). Resilience refers to the ability to endure difficult situations and gain strength from overcoming them (Fletcher and Sarkar, 2013). Resilience is a predictor of positive emotions (Philippe et al., 2009), which helps employees maintain their focus on work-related tasks (Ojo et al., 2021). Resilience refers to an individual's ability to combat feelings of hopelessness when encountering a difficult situation (Uppathampracha and Liu, 2022). Employee resilience is characterized as a dispositional variable in charge of the psychological mechanisms allowing employees to recover from difficult circumstances, traumatic incidents, and hardships (Kuntz et al., 2017). In other words, employee resilience acts as a protective factor in the responses of employees to changes and adjustments at work, enabling them to manage and recover from the frequent challenges or difficulties that are common in their professional setting (Okojie et al., 2023). Resilience manifests as an ability to bounce back from hardship (Avey et al., 2008). Hence, resilience is an important component in determining a person's abilities to cope with life's challenges (Zehir and Narcikara, 2016).

Scholars identified psychological resources, such as resilience, as instrumental to creativity and innovation at work (Amabile et al., 2004; Caniëls et al., 2022). Per Michaelis et al. (2010), resilient individuals have been shown to be more receptive to change and more innovative. Individuals who demonstrate strong resilience tend to achieve success in their innovative work behaviors (Cho and Lee, 2014). Bani-Melhem et al. (2021) highlighted that innovative work behavior is essential for cultivating employees who are both resilient and reliable. Additionally, de Weerd-Nederhof et al. (2018) observed that an individual's recognition of their own resilience can boost their ability to engage in innovative work behavior after encountering challenges. Malik (2022) suggest that the resilient capacity of employees encourage them to tackle workplace difficulties and consistently discover new methods to adapt to changes. Employee resilience fosters the development of new ideas and innovation by drawing on past experiences, leading to more effective work and adaptable changes in the future (Panpakdee and Limnirankul, 2018). Research indicates that resilient employees are more linclined to engage in disruptive creative behavior, particularly when faced with challenging work conditions (De Clercq and Pereira, 2019). Hence, the development of employee resilience contributes to the enhancement of workers' emotional, mental, and physical resources, which collectively form the basis of employee creativity (Sunley et al., 2019). Based on the provided arguments, this research suggests a notable connection among employee resilience, creativity, and innovation, as outlined in the following proposed hypotheses:

*H3: Employee resilience is significantly related to employee innovation*.
*H4: Employee resilience is significantly related to employee creativity*.

### 2.4 Job autonomy

Numerous studies have explored the factors contributing to innovation, yet the role of job autonomy has received limited attention. There remains a gap in empirical research examining the relationship between job autonomy and innovative behaviors (Swaroop and Dixit, 2018). Orth and Volmer (2017) suggested that job autonomy and its relationship with innovative work needs to be further explored. Autonomy, defined as the freedom to choose how work is executed, has long been considered a key antecedent to favorable employee outcomes in the workplace (Hackman and Oldham, 1975). Job autonomy is the degree to which a person has the independence and freedom to perform their assigned tasks (Zhang et al., 2017). When employees have autonomy, they experience a sense of self-determination and are not subject to external controls or limitations (Deci et al., 1989). Job autonomy involves the level of control employees have over choosing their tasks and determining the methods and timing for their completion (Orth and Volmer, 2017). When employees have the autonomy to carry out their tasks, they can discover and refine work methods that suit them best (De Spiegelaere et al., 2015). This kind of “space” is crucial for fostering creativity and innovative behavior, as these activities are centered on experimenting and finding the most effective ways to address problems (Purc and Laguna, 2019). Consequently, job autonomy can be seen as a vital job resource that enhances work engagement and creativity (Zhang et al., 2017).

Job autonomy plays a crucial role in fostering creativity and innovation in higher education. Faculty members with greater autonomy in their work methods demonstrate increased innovative behavior and performance (Dara, 2023). Job autonomy is probably connected to employee innovation as it helps to lessen the negative effects of dispositional resistance to change on innovative behavior (Battistelli et al., 2013) and promotes employee endorsement of organizational change (Hornung and Rousseau, 2007). The meta-analysis by Hammond et al. (2011) found that among the various factors they evaluated, job characteristics, especially job autonomy, emerged as the most crucial predictors of creativity and innovation. Putri and Prastika (2024) also reported a significant positive correlation between job autonomy and innovative work behavior of employees. As noted by Herentrey (2023), when employees feel they have more control over their tasks, they tend to be more engaged and innovative. This sense of autonomy not only enhances their work performance but also boosts their overall job satisfaction and wellbeing. Nevertheless, previous studies have indicated that job autonomy might not always serve as a beneficial resource (Zhang et al., 2017). A meta-analysis indicated that employees in roles with high levels of autonomy do not uniformly experience enhanced job attitudes, improved wellbeing, or consistently demonstrate positive work behaviors (Ng and Feldman, 2014). Based on the presented arguments, this research suggests a positive and significant correlation between job autonomy, creativity and innovation.

*H5: Job autonomy is significantly related to employee innovation*.
*H6: Job autonomy is significantly related to employee creativity*.

### 2.5 Mediating role of employee creativity

Over the past few decades, creativity and innovation have emerged as crucial abilities for achieving success in both emerging and established economies (Elrehail et al., 2018). Due to their close relationship in organizational contexts, these terms are often used interchangeably. However, there is disagreement among authors regarding the interaction between these concepts (Maulding, 2023). Some scholars advocate for more distinct definitions of creativity and innovation, viewing them as part of a two-phase process with specific characteristics. In this framework, creativity serves as the initial stage, encompassing the generation of ideas. Innovation follows as the second phase, involving the execution or sustenance of these creative concepts (De Vries et al., 2016). In other words, creativity involves generating new and valuable ideas by an individual or a small team collaborating, whereas innovation is about effectively putting these creative concepts into practice within a company (Amabile, 1988). Thus, it is highlighted that within organizations, creativity is the process of coming up with ideas, while innovation is the act of putting those ideas into practice (Morgan, 2020).

In a world characterized by constant change, individual creativity is becoming increasingly vital for successfully entering the job market, generating new ideas, and fostering innovation (Samašonok and Juškevičiene, 2022). Creativity is highly valued in modern organizations as a means to boost performance, allowing them to quickly respond to shifts in technological and environmental conditions (Choi et al., 2009). Moreover, in the current business landscape, where uncertainty and risk are on the rise, creativity is a crucial requirement for maintaining a competitive edge (Pattnaik and Sahoo, 2021). Creativity can be described as a cognitive ability that manifest as a novel or skill-based outcome, incorporates newly developed problem-solving methods, and utilizes a person's intelligence components to produce something uniquely original (Karaboga et al., 2022). According to Amabile and Pratt (2016), creativity involves generating innovative and valuable ideas to address a problem. Creativity in academia is essential and can be considered one of the most valued skills of all time (Benitez, 2024). Educators who possess creativity-relevant skills and abilities are valuable when they produce innovations in products, services, or processes (Collins and Cooke, 2013).

Existing research demonstrates the clear impact of creativity training on innovative outcomes (Børing, 2017; Liu et al., 2017), a relationship also suggested in Amabile (1988) theoretical framework. Numerous studies have extensively explored the association and determined that creativity positively influences innovation (Anderson et al., 2014; Hughes et al., 2018). It is clear that innovation relies on the creative efforts of individuals or employees (Chaubey et al., 2022). A creative idea acts as the cornerstone of innovation (Ghosh, 2015). Zare and Flinchbaugh (2019) proposed that creativity is best understood as the behavior of individual employees, which is crucial for driving innovation at the team or organizational level. In conclusion, creativity is linked to innovation and plays a crucial role in driving it, even though they might seem conceptually different (Chaubey et al., 2022).

**Figure 1:**
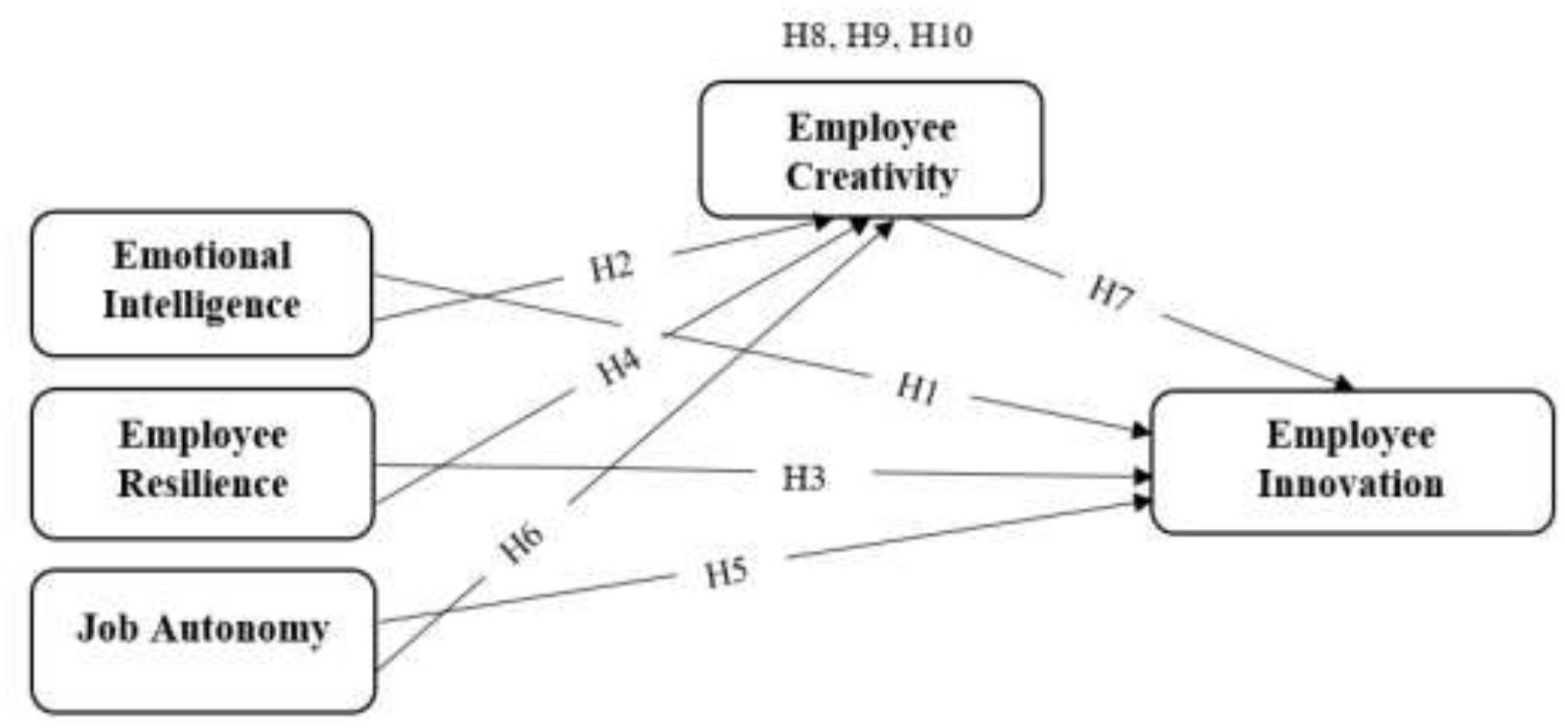
Hypothesis model.

As discussed in preceding sections, the connection between job autonomy, employee resilience, emotional intelligence and innovation is not always straightforward and is affected by several intervening factors. Research suggests that employee creativity plays a significant mediating role in various workplace relationships. Many scholarly investigations have delved into the role of creativity as an essential driver of innovation (Amabile, 1988; Jiang et al., 2014; Valgeirsdottir and Onarheim, 2017). The findings from the research conducted by Karaboga et al. (2022) demonstrated that employee creativity played a mediating role in connecting personal accomplishment to task performance. Creativity fully mediates the relationship between employee engagement and job performance, highlighting its importance in translating engagement into tangible results (Ismail et al., 2019). Similarly, training enhances organizational innovation via employee creativity, with organizational climate moderating this relationship (Chaubey et al., 2022). The theoretical framework and research model suggest a link between personal factors and creativity, as well as between creativity and innovation (Figure 1). This led us to contemplate that creativity might serve as a mediator between individual factors and innovative work behavior. The following hypotheses have been formulated based on the presented arguments:

*H7: Employee creativity is significantly related to employee innovation*.
*H8: Employee creativity mediate the relationship between emotional intelligence and employee innovation*.
*H9: Employee creativity mediate the relationship between employee resilience and employee innovation*.
*H10: Employee creativity mediate the relationship between job autonomy and employee innovation*.

## 3 Research methodology

### 3.1 Design

This research employs a quantitative cross-sectional method, which corresponds with the study's research framework (illustrated in Figure 1) and its objectives. Bryman (2006) suggests that a quantitative approach is the most suitable methodology, as it employs a survey design to gather numerical data and utilizes statistical techniques to examine the relationships between variables. A quantitative approach involves gathering numerical data, which is subsequently used for statistical analysis. This data is obtained from assessments conducted by individuals who are directly involved with the subject matter being examined (Al-Ajlouni, 2020). Consequently, the gathered data enables the findings to be generalized beyond the current study's sample (Sekaran and Bougie, 2016). This method, therefore, enhances comprehension of the underlying mechanisms of relationships and the associations between variables (Buchko, 1993).

### 3.2 Sampling and data collection

This research focused on analyzing full-time academic staff members at four universities located in South-Western Nigeria. The target population for this study comprised senior lecturers, associate professor and professor from both federal and state universities in the region. The selected institutions are considered first-generation universities, indicating that they were among the earliest established based on their institutional status. Convenience sampling was chosen for this research due to its accessibility and adaptability, which provided advantages for the study. The sample size for this research was determined using the Taro Yamane formula, ensuring a 95% confidence level (Yamane, 1973). Insert the numbers into the equation; the sample count is 307, yet the formula for sample size specifies the necessary number of responses. To accommodate individuals who are unreachable, it is common to increase the sample size by 30% to compensate for non-response (Yesuf et al., 2023). To ensure the data's reliability, the researchers expanded the sample size to 400 participants. They distributed 400 Google survey forms and received 329 completed responses. An additional 19 responses showed less than 20% missing data and exhibited a very low standard deviation. Upon closer examination, it was found that these participants had provided nearly identical answers to almost every survey question, rendering their responses of little value, and thus, they were excluded from further analysis. Ultimately, 310 questionnaires were fully completed without any missing data. The final sample (*N* = 310) is composed of 179 males and 131 females. The demographic information is presented in Table 1.

**Table 1:** Demographic information about academic staff (N = 310).

**Variable**	**Level**	**Frequency**	**Valid percentage**
Gender	Male	179	58
	Female	131	42
Age	Under 35 years	110	36
	35 to 50 years	126	40
Above 50 years	74	24
Education level	Higher National Diploma	18	6
	Bachelor's Degree	24	8
	Master's Degree	97	31
	Ph.D.	171	55
Employment status	Full-time	154	50
	Part-time	103	33
	Not specified	53	17
Years with organization	Under 5 years	103	33
	6–10 years	112	36
	11–20 years	73	23
	21 years and above	22	8
Academic rank	Professor	63	20
	Ass. professor	110	35
	Senior lecturer	76	25
	Lecturer	61	20
	Total	310	100

Participants were instructed to fill out the questionnaire without disclosing their identities, and the data was collected through a trusted online survey tool, specifically Google Forms. The advantages of online surveys vs. other types of surveys include convenience and perceived confidentiality (Regmi et al., 2016). Within the field of social sciences, employing surveys is a common strategy, often associated with a deductive approach to conducting research (Rahi, 2017). Utilizing a questionnaire is deemed the most suitable technique for gathering data in this study, as it facilitates obtaining responses from a large number of employees swiftly (Rahi, 2017; Rowley, 2014). The data acquired can be analyzed to produce results that are more broadly generalizable (Yesuf et al., 2023). Each faculty's dean received an online survey link to distribute via email to the university's academic staff members. The survey was optional for participants, and the study received approval from the researchers' Institutional Review Board (IRB). The survey was disseminated between April 2023 and June 2023. This survey aims to explore how university staff members perceive their capacity for creativity and innovation in their work environment.

### 3.3 Analysis tool

For the statistical analysis in this research, data coding and assumption verification were conducted using the Social Package for Social Science (SPSS, v.26). Structural equation modeling (SEM) was performed with SMART Partial Least Squares (PLS 4), facilitating the validation of the measurement model and the analysis of path estimation. Recently, PLS has emerged as the most popular method for multivariate analysis across various fields (Al-Ajlouni, 2020). PLS-SEM, a variance-based approach in structural equation modeling, was utilized due to its strength and the exploratory nature of the study (Fauzi et al., 2019). A two-step model encompassing both measurement and structural aspects was proposed and identified as the standard for presenting PLS-SEM results (Chin, 2009). The model's complexity, which assesses 10 relationships among the variables, serves as another rationale for employing PLS-SEM (Fauzi et al., 2019).

### 3.4 Items generation

The questionnaire items were adapted from previous research and tailored to fit the study's scope. Since the measurement instrument's items were derived from previous studies, ensuring their validity and reliability was essential (Farrukh et al., 2021). To ensure the questionnaire's face validity, the researchers engaged with scholars from Nigerian universities. This study implemented a pilot test with 30 respondents to verify the statistical validity and reliability of the instrument. The analysis showed that all constructs had outstanding internal consistency, with Cronbach's alpha values above 0.90 (Table 2). Some items were slightly reworded to improve clarity and better fit the context. These changes were included in the final questionnaire to further enhance its validity and reliability. The survey was structured into six parts, with the initial section focusing on demographic data. This first segment included four questions designed to differentiate among participants, covering aspects such as gender, qualification, position, years of working. In this research, five factors were measured using validated and reliable instruments. Table 1 provides a description of the operational definitions for each construct, along with the number of items and the source of adaptation. Every metric was evaluated on a 5-point Likert scale (1 = strongly disagree, 2 = disagree, 3 = neutral, 4 = agree, 5 = strongly agree).

**Table 2:** Construct operational definition and source adaptation.

**Construct**	**Operational definition**	**No. of items**	**Source adaption**
Innovation	Innovation is effective implementation of creative ideas within the university	6	(Scott and Bruce, 1994)
Creativity	Creativity involves the generation of novel and beneficial ideas by either an individual or a small collaborative group.	13	(Nasifoglu Elidemir et al., 2020)
Emotional intelligence	Emotional intelligence (EI) refers to the capacity to recognize, comprehend, utilize, and regulate emotions in oneself and others.	14	(Palmer et al., 2009)
Employee resilience	Employee resilience encompasses a range of proactive and adaptive behaviors that promote change and innovation while enhancing employee wellbeing.	6	(Al-Omar et al., 2019)
Job autonomy	Job autonomy encompasses the latitude given to employees within university, enabling them to work with flexibility and potentially spark innovative approaches.	9	(Lee, 2018)

The survey instrument comprises 48 items in total. A 6-item scale (Scott and Bruce, 1994) was utilized to assess innovation, with an example item being “I am able to search for new working methods, techniques or instruments”. Creativity was evaluated using a 13-item scale from (Nasifoglu Elidemir et al., 2020), which includes items such as “I suggest new ways of performing work tasks”. Additionally, emotional intelligence was gauged using a 14-item scale from (Palmer et al., 2009), featuring items like “I appropriately communicate decisions to stakeholders”. A 6-item scale from (Al-Omar et al., 2019) was employed to measure employee resilience, with a sample item stating “I usually come through difficult times with little trouble”. Finally, job autonomy was assessed using a 9-item scale (Lee, 2018), which includes items such as “The job allows me to make decisions about what methods I use to complete my work”. This survey is being conducted to explore how academic members at universities perceive their own abilities to be creative and innovative within their professional settings.

## 4 Analysis and results

This study employed PLS-SEM path modeling to evaluate the proposed theoretical model. This approach was chosen for several reasons. First, it is widely utilized and has seen extensive application in management and related fields (Umrani et al., 2024). Second, given that the study's objective is to analyze the outcome variable, the PLS path was deemed an appropriate method (Hair et al., 2019a). Finally, this approach is considered the most advanced and commonly used (Umrani et al., 2024). As a result, Smart PLS 4 was utilized for this research. This research utilizes a dual-phase method: initially assessing the measurement model, followed by an evaluation of the structural model (Henseler et al., 2009; Osman et al., 2024).

### 4.1 Measurement model assessment

**Figure 2:**
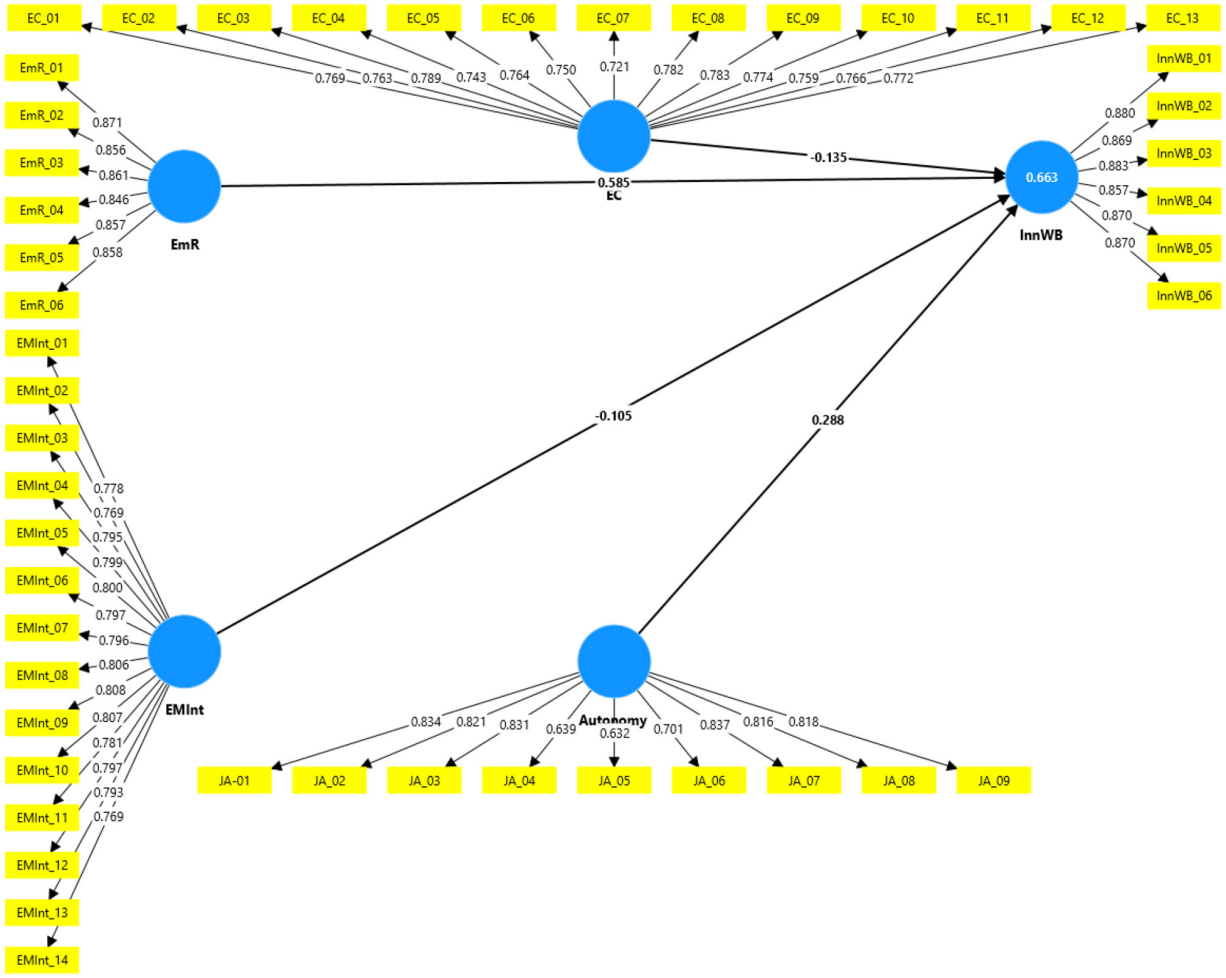
Measurement model.

In accordance with Hair Jr et al. (2023) and Henseler et al. (2009), researchers must evaluate the measurement model by examining several key factors (Figure 2). These include the individual reliability of each item, the internal consistency, content validity, and both convergent and discriminant validity. This assessment process is crucial for ensuring the robustness of the research methodology.

### 4.2 Individual item reliability

Hair et al. (2017) and Duarte and Raposo (2010) recommended to evaluate the reliability of individual items by analyzing the outer loadings of each measure across all constructs. Additionally, there is a general guideline to keep items with reliability scores between 0.40 and 0.70. Consistent with this, the outer loadings of all items in this study are observed to be 0.5 or higher (see Table 1), indicating that our study meets the criteria for acceptable item reliability.

### 4.3 Internal consistency reliability

As stated by Bagozzi and Yi (1988) and Hair et al. (2019b), there is an established guideline for interpreting the composite reliability (CR) coefficient, suggesting it should be 0.7 or higher. The coefficients of CR (rho_a, rho_c) and Cronbach Alpha are given in Table 3. These values have acceptable consistency.

**Table 3:** Measurement model results.

**Construct**	**Items**	**Loadings**	**Alpha**	**CR(rho_a)**	**CR(rho_c)**	**AVE**
Employee creativity	EC_01	0.771	0.941	0.941	0.948	0.584
	EC_02	0.762				
	EC_03	0.788				
	EC_04	0.744				
	EC_05	0.767				
	EC_06	0.749				
	EC_07	0.715				
	EC_08	0.779				
	EC_09	0.785				
	EC_10	0.776				
	EC_11	0.756				
	EC_12	0.772				
	EC_13	0.773				
Emotional intelligence	EMInt_01	0.778	0.954	0.955	0.959	0.628
	EMInt_02	0.768				
	EMInt_03	0.796				
	EMInt_04	0.799				
	EMInt_05	0.800				
	EMInt_06	0.798				
	EMInt_07	0.795				
	EMInt_08	0.805				
	EMInt_09	0.809				
	EMInt_10	0.808				
	EMInt_11	0.781				
	EMInt_12	0.796				
	EMInt_13	0.793				
	EMInt_14	0.768				
Employee resilience	EmR_01	0.870	0.928	0.929	0.944	0.736
	EmR_02	0.856				
	EmR_03	0.859				
	EmR_04	0.843				
	EmR_05	0.860				
	EmR_06	0.860				
Innovative work behavior	InnWB_01	0.879	0.937	0.937	0.950	0.760
	InnWB_02	0.872				
	InnWB_03	0.883				
	InnWB_04	0.859				
	InnWB_05	0.869				
	InnWB_06	0.867				
Job autonomy	JA-01	0.838	0.915	0.933	0.930	0.599
	JA_02	0.816				
	JA_03	0.832				
	JA_04	0.623				
	JA_05	0.620				
	JA_06	0.686				
	JA_07	0.839				
	JA_08	0.830				
	JA_09	0.830				

### 4.4 Convergent validity

This research focused on assessing convergent validity to determine AVE values. According to Henseler et al. (2015), an AVE value of 0.50 or higher indicates that at least 50% of the indicator variance is explained. In this study, all AVE values exceeded the 0.50 threshold, confirming convergent validity.

### 4.5 Discriminant validity through Fornell-Larcker

This study assessed the Fornell–Larcker ratio to test discriminant validity (Fornell and Larcker, 1981). According to Table 4, the Fornell and Larcker test values exceed the correlations between the variables.

**Table 4:** Discriminant validity through Fornell-Larcker.

	**Autonomy**	**EC**	**EMInt**	**EmR**	**InnWB**
Autonomy	0.773				
EC	0.751	0.765			
EMInt	−0.755	−0.545	0.793		
EmR	0.724	0.763	−0.647	0.858	
InnWB	0.747	0.580	−0.630	0.786	0.872

### 4.6 Discriminant validity through Hetrotrait-Monotrait ratio

This research employed the Hetrotrait-Monotrait (HTMT) ratio, following the recommendations of Henseler et al. (2015). According to their criterion, the HTMT values fell below the 0.90 threshold (refer to Table 5 for specific values). Consequently, discriminant validity was not identified as an issue in this investigation. These findings substantiate the discriminant validity of the current study.

**Table 5:** Discriminant validity (HTMT).

**Variables**	**Autonomy**	**EC**	**EMInt**	**EmR**	**InnWB**
Autonomy					
EC	0.785				
EMInt	0.824	0.575			
EmR	0.882	0.816	0.687		
InnWB	0.797	0.618	0.664	0.843	

### 4.7 Multicollinearity

This study assessed the issue of multicollinearity in the data using the variance inflation factor (VIF). Becker et al. (2015) recommended that the values of VIF must be < 5. This study observed VIF values that fell within this recommended range, indicating that there is no multicollinearity issue in the data (Table 6).

**Table 6:** Collinearity statistics (VIF values).

	**VIF**		**VIF**		**VIF**
EC_01	2.180	EMInt_04	2.582	EmR_06	2.717
EC_02	2.097	EMInt_05	2.524	InnWB_01	3.157
EC_03	2.289	EMInt_06	2.510	InnWB_02	2.899
EC_04	1.993	EMInt_07	2.491	InnWB_03	3.171
EC_05	2.182	EMInt_08	2.628	InnWB_04	2.751
EC_06	2.060	EMInt_09	2.597	InnWB_05	2.946
EC_07	1.808	EMInt_10	2.596	InnWB_06	2.933
EC_08	2.217	EMInt_11	2.361	JA-01	2.777
EC_09	2.290	EMInt_12	2.450	JA_02	2.459
EC_10	2.200	EMInt_13	2.498	JA_03	2.719
EC_11	2.055	EMInt_14	2.174	JA_04	1.889
EC_12	2.218	EmR_01	2.904	JA_05	1.682
EC_13	2.205	EmR_02	2.649	JA_06	2.063
EMInt_01	2.274	EmR_03	2.715	JA_07	2.592
EMInt_02	2.229	EmR_04	2.518	JA_08	2.464
EMInt_03	2.492	EmR_05	2.666	JA_09	2.506

### 4.8 Structural equation modeling

**Figure 3:**
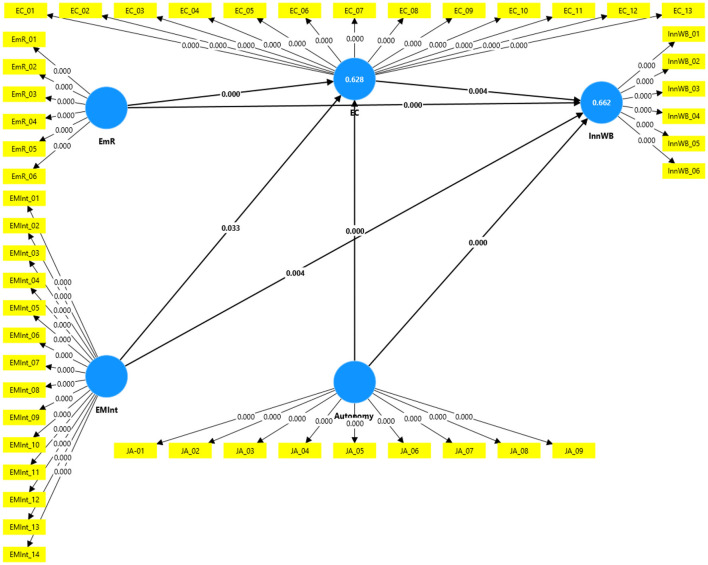
Structural model.

After validating the measurement model, the structural model was assessed to examine underlying relationships (Figure 3). The model's fit was evaluated using the standardized root mean square residual (SRMR), which should be below 0.08 for samples exceeding 100 (Henseler et al., 2009). This study demonstrated a significant model fit (0.042). Endogenous latent variables with coefficients of determination (*R*^2^) of 0.662 and 0.628 can be categorized as substantial (Hair et al., 2010, 2019b). Figure 2 indicates *R*^2^ (Creativity) = 0.628 and *R*^2^ (Innovation) = 0.662, suggesting the structural model possessed satisfactory in-sample predictive power, aligning with previous research in this field (Yesuf et al., 2023). Additionally, the Q^2^ value should exceed zero (Creativity = 0.620) and (Innovation = 0.649). The findings of this research fell within the significance threshold, confirming the model's predictive validity (Falk and Miller, 1992). Additionally, the researchers assessed the magnitude and significance of the path coefficients that represent the hypotheses. To determine the significance of these coefficients, they employed a bootstrapping technique (utilizing 5,000 samples). The outcomes of the structural model are depicted in Figure 2. A comprehensive overview of the path coefficients, along with their standard deviations, *t*-statistics, and *p*-values, is presented in Table 7.

**Table 7:** Path coefficients.

	**Beta**	**STDEV**	***t*-value**	***P* values**	**Decision**
Autonomy -> EC	0.420	0.049	8.513	0.000	Supported
Autonomy -> InnWB	0.289	0.059	4.919	0.000	Supported
EC -> InnWB	−0.141	0.049	2.885	0.004	Supported
EMInt -> EC	0.075	0.035	2.132	0.033	Supported
EMInt -> InnWB	−0.107	0.037	2.879	0.004	Supported
EmR -> EC	0.466	0.051	9.133	0.000	Supported
EmR -> InnWB	0.587	0.045	13.159	0.000	Supported

According to the PLS–SEM findings, (H1) testing the direct effects of emotional intelligence on employee innovation revealed a significant relationship (β = −0.107, *t* = 2.879, *p* = 0.004). H2 found a significant relationship between emotional intelligence and employee creativity (β = 0.075, *t* = 2.132, *p* = 0.033). Thus, H1 and H2 were supported. Further, H3 and H4 indicated that employee resilience has a positive and significant influence on employee innovation and creativity (β = 0.587, *t* = 13.159, *p* = 0.000); (β = 0.466, *t* = 9.133, *p* = 0.000). These results thus confirm hypotheses H3 and H4. The findings demonstrated that job autonomy has a significant influence on employee innovation and creativity (β = 0.289, *t* = 4.919, *p* = 0.000); (β = 0.420, *t* = 8.513, *p* = 0.000). Thus, both H4 and H6 were supported. Finally, H7 found a significant relationship between employee creativity and employee innovation (β = −0.141, *t* = 2.885, *p* = 0.004). This thus confirms hypothesis H7.

In terms of mediating effects, there was a significant indirect effects of employee resilience on employee innovation (β = −0.066, *t* = 2.746, *p* = 0.006) via employee creativity. Therefore, it was concluded that employee creativity partially mediated the relationships between employee resilience and employee innovation. Thus, H9 was supported. Further, the results indicated a significant indirect effects of job autonomy on employee innovation through employee creativity (β = −0.059, *t* = 2.715, *p* = 0.007), which shows partial mediator in the model (Table 8). Thus, H10 was supported. However, the results showed no indirect effects of emotional intelligence via employee creativity on employee innovation (β = −0.011, *t* = 1.566, *p* = 0.118). Hence, H8 was not supported.

**Table 8:** Hypothesis constructs.

**Total effects (JA-**>**Inn)**			**Direct effect (JA-**>**Inn)**			**Hypothesis**	**Indirect effect (JA-**>**Inn)**			
Coefficient	*t* value	*p*-value	Coefficient	*T* value	*p*-value	JA->EC->Inn	Coefficient	SE	*t* value	*p*-value
0.230	4.168	0.000	0.289	4.919	0.000		−0.059	0.022	2.715	0.007
Total effects (EMInt->Inn)			Direct effect (EMInt ->Inn)				Indirect effect (EMInt ->Inn)			
Coefficient	*t* value	*p*-value	Coefficient	*T* value	*p*-value	EMInt ->EC->Inn	Coefficient	SE	*t* value	*p*-value
−0.117	3.007	0.003	−0.107	2.879	0.004		−0.011	0.007	1.566	0.118
Total effects (EmR->Inn)			Direct effect (EmR->Inn)			Hypothesis	Indirect effect (EmR->Inn)			
Coefficient	*t* value	*p*-value	Coefficient	*T* value	*p*-value	EmR->EC->Inn	Coefficient	SE	*t* value	*p*-value
0.521	10.675	0.000	0.587	13.159	0.000		−0.066	0.024	2.746	0.006

## 5 Discussion

This research provides theoretical and practical implications for the higher education sector, highlighting the significance of job autonomy, staff resilience, and emotional intelligence in fostering innovation among academics. By applying SEM, this study examined the mediating effect of creativity on the relationship between HPWS and IWB in Nigerian higher education. This research fills a critical gap in the literature, as few studies have empirically tested the role of employee creativity as a mediator in this context. The research underscores the critical significance of faculty members' creative abilities in fostering innovation within Nigerian higher education institutions. This survey seeks to make a contribution to these research areas. In general, the quantitative, cross-sectional study results help to elucidate the roles of job autonomy, employee resilience, and emotional intelligence in fostering employee innovation. This study observed that employee creativity partially mediates the relationship between job autonomy, employee resilience, emotional intelligence and employee innovation.

Over the past two decades, there has been a dearth of studies examining the impact of emotional intelligence on creativity and innovation (Dasgupta, 2023). This gap in research presents significant opportunities for scholars to delve into this field of study. The findings of this research demonstrate that employee intelligence plays a crucial role in fostering innovation. These results align with earlier investigations (Al-Omari, 2017; Hu and He, 2018), which explored various sectors, whilst the current study concentrated on higher education institutions (HEIs). Malik (2021) suggests that employees with high emotional intelligence direct their emotional energy toward fostering innovation at work by coming up with novel ideas or methods, advocating for these concepts within their organizations, and ultimately implementing them to gain advantages. Additionally, studies show that EI helps professionals in HEIs to become more knowledge-focused, autonomous, accountable, and creative in their daily tasks (Shafait et al., 2021). However, failing to recognize how emotional intelligence (EI) can be utilized by institutions to foster creativity and innovation may result in an unenthusiastic workforce, unhappy clients, and a decline in overall organizational effectiveness (Dasgupta, 2023).

This research represents an initial exploration into how resilience, as an aspect of personality, affects the innovative behavior of academic staff through workplace creativity. Utilizing the Dynamic Componential Model of Creativity and Innovation as a foundation, this research constructs a mediational framework wherein employee creativity serves as an intermediary between employee resilience and innovative behavior. Our findings align with previous research suggesting a connection between resilience and innovative work behavior (Caniëls et al., 2022; Cho and Lee, 2014). We extend this body of knowledge by demonstrating that employee resilience is a predictor of creativity, which subsequently forecasts innovative work behavior (IWB). This conclusion is supported by the outcomes of our tests for Hypotheses 3 and 4. Khahan et al. (2024), suggest that employees who possess resilience tend to demonstrate better behavioral outcomes, as resilience is a beneficial psychological factor that affects other positive factors. However, employee resilience is a crucial skill that enables individuals to tackle challenges, thereby boosting their confidence and creativity. When employees lack resilience, particularly during periods of upheaval or transition, it can result in higher turnover rates, lack of productivity, and reduced overall employee wellness (Anderson, 2024).

The role of autonomy in fostering creativity and innovation is widely recognized. Employee autonomy refers to the freedom to operate conveniently for them, allowing for the possibility of innovation (Suhandiah et al., 2023). Freedom from outside pressures allows individuals to focus on the inherent value of their work, increases intrinsic motivation and thereby promoting innovative behavior (Li and Zhu, 2022). In line with previous studies, autonomy is identified as an element of human motivation that has the capacity to stimulate creative activities (Beirne et al., 2017; Dixit and Upadhyay, 2021; Lifshitz-Assaf et al., 2019; Noble-Nkrumah et al., 2022; Zhang et al., 2017). Moreover, employees who have greater autonomy in their roles are more inclined to employ innovative behaviors when tackling workplace challenges (Orth and Volmer, 2017; Suhandiah et al., 2023; Swaroop and Dixit, 2018). Research indicates that the autonomy instructors experience in their work, alongside other occupational resources, shows a positive relationship with their weekly engagement levels, which subsequently enhances their job performance (Bakker and Bal, 2010). Furthermore, the degree of autonomy in teaching roles plays a part in fostering collective innovation among educators by facilitating their involvement in professional development activities (Nguyen et al., 2021). Allowing staff members to choose their own methods for completing tasks may enhance their motivation to create and adopt innovative ideas and technologies (Li and Zhu, 2022). To foster innovation, organizations need to effectively manage and encourage job autonomy that nurtures the innovative behaviors of their employees (Alpkan et al., 2010; Dobni, 2010).

The study's results for discriminant validity, based on the Fornell-Larcker criterion, show that the correlation between Autonomy and Employee Resilience (0.824) exceeds the square root of the AVE for Autonomy (0.773). However, this concern is addressed by the Heterotrait-Monotrait (HTMT) ratio results, where all construct pairs, including Autonomy and Employee Resilience, fall below the recommended threshold, indicating adequate discriminant validity. Recent methodological research (e.g., Henseler et al., 2015) highlights that HTMT is a more reliable and sensitive measure of discriminant validity compared to the traditional Fornell-Larcker criterion, which can underestimate discriminant validity in certain cases. Consequently, prioritizing HTMT as the main assessment criterion is warranted, as it offers a more rigorous evaluation of construct distinctiveness, thereby reinforcing confidence in the measurement model and subsequent structural analyses.

The structural model unexpectedly indicated a negative association between Employee Creativity and Innovation (β = −0.141), which runs counter to widely accepted theories that view creativity as a primary driver of innovation. This counterintuitive result may be due to the nuanced, mediating function that creativity plays within the model. Although factors like Job Autonomy, Emotional Intelligence, and Employee Resilience tend to enhance creativity, the transformation of creative ideas into innovative practices can be obstructed by organizational or contextual challenges—such as insufficient resources, a culture of risk aversion, or resistance from management. Additionally, when creativity is not supported by appropriate organizational mechanisms, it may lead to frustration or a sense of idea fatigue, ultimately hampering innovation. These results underscore the importance of examining the broader organizational context and the enabling factors that facilitate the translation of creativity into innovation. Future research should focus on identifying and understanding these contextual moderators to clarify under what circumstances employee creativity most effectively leads to innovative outcomes.

The analysis confirms a significant and positive direct impact of emotional intelligence on innovative work behaviors; however, the mediation analysis showed that employee creativity does not significantly mediate this relationship (Hypothesis 8). This surprising result could be explained by various contextual or cultural factors. In certain organizational environments—especially those with hierarchical structures or low psychological safety—employees might have high emotional intelligence but still feel limited in expressing creativity due to strict rules or fear of failure. Moreover, in collectivist cultures, employees may emphasize conformity and group harmony over individual creative expression, which could reduce the mediating effect of creativity. These findings indicate that although emotionally intelligent employees can directly drive innovation, organizational culture and climate may restrict creativity's role as a conduit to innovation. Further qualitative research could provide deeper insights into these dynamics.

## 6 Implications

### 6.1 Theoretical implications

The study's results provide valuable theoretical insights into the impact of personal and work environmental factors on creative and innovative outcomes within higher education contexts. This understanding is rooted in The Dynamic Componential Model of Creativity and Innovation (Amabile and Pratt, 2016), which serves as the theoretical foundation for the research. This research appears to be the first to explicitly examine the combined effects of personal and work-related factors such as emotional intelligence, employee resilience, and job autonomy on innovative behavior of academic staff in Nigeria. The finding of this study extend the vein of research by examining the role of employee creativity as a mediator in these relationship. The Dynamic Componential Model of Creativity and Innovation (Amabile and Pratt, 2016) supports this mediation model by demonstrating that personal factors influence creative output, which in turn drives innovative behavior. Therefore, it provides a strong theoretical foundation for this research framework.

In today's competitive landscape, universities must embrace innovation. Universities operating in such an environment need dynamic staff members who are prepared to exceed their basic job requirements and do not have to be induced or controlled for such extra-role behaviors (Swaroop and Dixit, 2018). Academic staff's ability to innovate in teaching, research, and creative endeavors is significantly influenced by their level of job autonomy. This study demonstrates that increased independence in the workplace promotes intrinsic motivation. As a result, this enhanced motivation leads to improved creative output and innovative practices among academic professionals. Moreover, faculty members with enhanced emotional intelligence are more adept at coping with stress, finding innovative solutions to problems, and working together to share knowledge, all of which enhance innovation in both teaching and research practices. Consequently, this study is expected to help universities and colleges identify various strategies needed to support their efforts in enhancing the emotional intelligence (EI) skills of their staff, with the ultimate goal of promoting innovation and creativity.

In addition to our theoretical contributions, this research enhances existing studies on resilience. Employee Resilience functions as a psychological asset that enables academics to surmount obstacles, maintain creative efforts, and adjust to evolving educational environments. Our results reinforce the idea that resilience mitigates stress and uncertainty, enabling faculty to stay involved in creative and innovative activities despite challenging work conditions. By combining these elements, our study expands the Dynamic Componential Model of Creativity and Innovation (Amabile and Pratt, 2016), showing how personal factors (emotional intelligence, resilience) and work-related aspects (job autonomy) interact to promote creativity and innovation in academic settings.

### 6.2 Practical implications

Theoretical advancements and empirical research are pertinent to practical applications. Universities aiming to enhance their staff's innovative work behavior should focus not only on hiring and retaining resilient individuals but also on fostering resilience among their employees. Resilience is a complex trait with characteristics similar to a state, meaning it can be gradually cultivated and enhanced over time (Caniëls et al., 2022). This research offers valuable insights for professionals regarding the significance of emotional intelligence in fostering innovative work behavior among academic staff in Nigerian higher education institutions. Universities should prioritize leveraging their employees' emotional intelligence levels, as it was demonstrated to have a substantial impact on their innovative work behavior. To accomplish this objective, institutions can implement specialized emotional intelligence training and mindfulness programs. These initiatives should concentrate on developing emotional competencies to cultivate an emotionally intelligent workforce. Additionally, organizations can enhance their recruitment strategies by incorporating emotional intelligence assessments and behavioral interview techniques, complementing the existing rigorous technical interviews, to attract and select emotionally intelligent candidates. Additionally, the study revealed that job autonomy positively influences IWB, indicating that teachers produce high-quality research when given independence in their work. Consequently, educational institutions aiming to maintain or achieve top rankings should prioritize granting job autonomy to their faculty members.

### 6.3 Limitations and future research

This study offers valuable insights into how emotional intelligence, employee resilience, and job autonomy influence innovative work behavior through creativity. However, it has certain constraints. Firstly, the study mainly examined academic personnel in public sector universities, potentially yielding outcomes that differ from those of private institutions in South-Western Nigeria. Consequently, these results are most relevant to faculty in public universities and may not be generalizable to private colleges. Subsequent research could explore how these variables affect innovative work behavior among academics in private higher education institutions across various regions of Nigeria. Secondly, given the study's exploratory nature, employing a quantitative deductive approach with a cross-sectional timeframe was deemed suitable. However, future studies should consider adopting a longitudinal design for data collection to examine the proposed relationships. This approach would provide a more comprehensive understanding of the causal connections among the variables. Additionally, there remains an opportunity for researchers to utilize qualitative methods to conduct in-depth investigations and explore how emotional intelligence, resilience, and autonomy drive creativity and innovation. Thirdly, this research employed a single data collection method (survey) and relied solely on employee self-assessment as the data source. Consequently, it should not be used to draw conclusions about causal relationships between variables.

## Data Availability

The raw data supporting the conclusions of this article will be made available by the authors, without undue reservation.
